# Divergent understandings in comparative oncology

**DOI:** 10.1073/pnas.2532925123

**Published:** 2026-02-02

**Authors:** Zachary T. Compton, Amy M. Boddy, Lisa M. Abegglen, Daniel Chávez, Joshua D. Schiffman, Marc Tollis, Carlo C. Maley

**Affiliations:** ^a^Arizona Cancer Evolution Center, The Biodesign Institute, Arizona State University, Tempe, AZ 85281; ^b^University of Arizona Cancer Center, Tucson, AZ 85724; ^c^University of Arizona College of Medicine, Tucson, AZ 85724; ^d^University of California, Santa Barbara, CA 93106; ^e^Department of Pediatrics, University of Utah and Huntsman Cancer Institute, Salt Lake City, UT 84112; ^f^Centro de Investigación de la Biodiversidad y Cambio Climático, Facultad deCiencias de Medio Ambiente, Universidad Tecnológica Indoamérica, Machala y Sabanilla, Quito EC170301, Ecuador; ^g^School of Informatics, Computing and Cyber Systems, Northern Arizona University, Flagstaff, AZ 86011; ^h^Biodesign Center for Biocomputing, Security and Society, Arizona State University, Tempe, AZ 85281; ^i^School of Life Sciences, Arizona State University, Tempe, AZ 85281

**Fig. 1.**
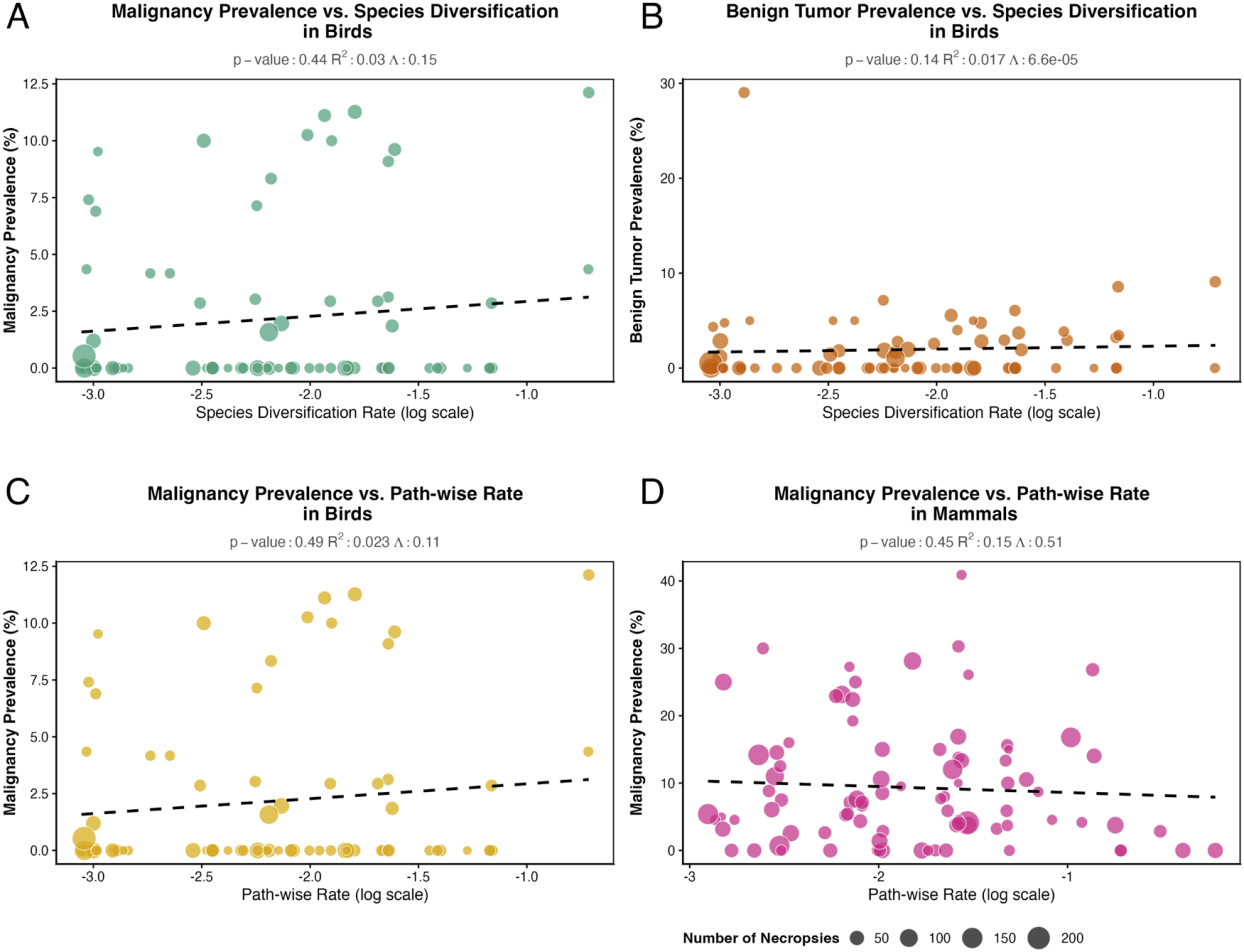
The four models reported by Butler et al. to be significant predictors of either benign tumor or malignancy prevalence are not statistically significant when analyzed with the phylogenetic regressions described in Compton et al. We used an arcsine-square root transform of the prevalence data, since otherwise it is constrained to the unit interval with many values near zero. (*A*) A nonsignificant relationship between species diversification rates and malignancy prevalence in birds. (*B*) A nonsignificant relationship between species diversification rates and benign tumor prevalence in birds. (*C*) A nonsignificant relationship between the path-wise evolutionary rate of body size and malignancy prevalence in birds. (*D*) A nonsignificant relationship between the path-wise evolutionary rate of body size and malignancy prevalence in mammals.

Butler et al.’s recent exploration of the rates of species diversification and cancer is an important addition to the study of comparative oncology (1). Do lineages that tend to diversify more rapidly get more cancer? The authors hypothesize that a susceptibility to genomic rearrangements might lead to higher diversification and cancer rates. However, because deleterious rearrangements are swiftly eliminated, only lineages with robust, stabilized genomes can diversify (2). Diversification is a signature of genomic stability rather than instability. Therefore, species with more stable genomes might diversify more and have less cancer. Unfortunately, the statistical model, previously published in Butler et al.’s analysis of body size and cancer (3), is deeply flawed. Butler et al. treat tumor counts as additive, therefore, any apparent relationship between diversification and cancer prevalence may simply reflect differences in sample size rather than related to evolution. Their approach estimates a proportion of necropies that will have cancer, which implies that all species have the same rate of cancer. The species differences only come through their differences in the other predictors (body size, change in body size, and diversification rate) which add or subtract a fixed number of cancers, regardless of the number of necropsies observed. With only 87 mammal and 76 bird species in their analysis, Butler et al. may lack the statistical power needed to detect interactions between the number of necropsies and their other predictors (they do not report testing for those interactions). An alternative statistical model allows each species to have a different intrinsic rate of cancer which is then modified by the other predictors like body size or diversification rate. When we reanalyzed their data under these assumptions using methods from Compton et al. (4), we found no evidence of an association between diversification rate and either malignancy or benign tumor prevalence in either mammals or birds (Fig. 1). Nor did we find an association between change in body size and malignancy in either birds or mammals (Fig. 1). While a larger sample size may prove such a relationship in the future, it is unlikely to rescue fundamentally flawed methodology.

While Butler et al.’s analysis of the rate of change in body size is intriguing, we predict that the direction of that change is important (5). Their analysis fails to distinguish between lineages that have recently shrunk versus recently grown. We predict these scenarios have opposite implications: shrinking lineages inherit robust cancer suppression from larger ancestors, while growing lineages could remain vulnerable to cancer until selection generates mechanisms appropriate to their new size (6). We appreciate that our colleagues have utilized the cancer prevalence data we published in innovative ways, but we are acutely aware of the need for many more data points to detect subtle effects and interactions between factors.
